# Impact of baseline and trajectory of the atherogenic index of plasma on incident diabetic kidney disease and retinopathy in participants with type 2 diabetes: a longitudinal cohort study

**DOI:** 10.1186/s12944-024-02003-5

**Published:** 2024-01-11

**Authors:** Jia Zhang, Cong Liu, Ying Peng, Qianhua Fang, Xing Wei, Cui Zhang, Lin Sun, Zhuomeng Hu, Jie Hong, Weiqiong Gu, Weiqing Wang, Juan Shi, Yifei Zhang

**Affiliations:** 1 Department of Endocrine and Metabolic Diseases, Shanghai Institute of Endocrine and Metabolic Diseases, Ruijin Hospital, Shanghai Jiao Tong University School of Medicine, 197 RuiJin Er Road, Shanghai, China; 2 Shanghai National Clinical Research Center for Metabolic Diseases, Key Laboratory for Endocrine and Metabolic Diseases of the National Health Commission of the PR China, Shanghai Key Laboratory for Endocrine Tumor, State Key Laboratory of Medical Genomics, Ruijin Hospital, Shanghai Jiao Tong University School of Medicine, Shanghai, China

**Keywords:** Type 2 diabetes, Trajectory, Diabetic retinopathy, Diabetic kidney disease, Atherogenic index of plasma, Lipid profile

## Abstract

**Background:**

Some studies have assessed the predictive role of the atherogenic index of plasma (AIP) for macrovascular diseases. This prospective investigation aimed to elucidate whether AIP is associated with diabetic kidney disease (DKD) and diabetic retinopathy (DR) incidence.

**Methods:**

The data were extracted from 4831 participants, of whom 2943 and 3360 participants with type 2 diabetes (T2D) were included in the DKD and DR follow-up analyses, respectively. Cox regression models were performed to test the relationships of AIP value at baseline with the risk of incident DKD and DR. Group-based trajectory modelling was utilized to discern AIP trajectories during the follow-up period. Subsequently, logistic regressions were applied to ascertain the influence of AIP trajectories on the incidence of DKD and DR.

**Results:**

During the follow-up period, 709 (24.1%) and 193 (5.7%) participants developed DKD and DR, respectively. The median (interquartile range) follow-up time was 24.2 (26.3) months for DKD and 25.7 (27.0) months for DR. According to the multivariate Cox regression models, baseline AIP was positively and linearly related to the occurrence of DKD, with a hazard ratio of 1.75 (95% confidence interval [CI] 1.36–2.26). Three distinct trajectories of AIP were identified throughout the follow-up time: Low (31.4%), Median (50.2%), and High (18.3%). Compared to participants with the Low AIP trajectory, those with High and Median AIP trajectories presented 117% (95% CI: 1.62–2.91) and 84% (95% CI 1.46–2.32) greater odds of developing DKD, respectively. However, neither baseline levels nor trajectories of AIP were shown to be related to DR after adjusting for confounding factors.

**Conclusions:**

Baseline levels and trajectories of AIP were independently related to elevated DKD risk, indicating that AIP could be used as a predictor for identifying T2D participants at higher risk of DKD. No association between AIP and DR was detected.

**Supplementary Information:**

The online version contains supplementary material available at 10.1186/s12944-024-02003-5.

## Background

Type 2 diabetes (T2D) has become a serious worldwide health challenge with a growing prevalence [1]. The primary cause of mortality in diabetic patients is chronic vascular complications [2], which can affect life expectancy and impose financial burdens [3]. These vascular complications are influenced by metabolic factors, of which dyslipidaemia is vital [4]. Notably, patients with diabetes exhibit a typical dyslipidaemia pattern characterized by elevated triglycerides (TG), reduced high-density lipoprotein cholesterol (HDL-C), and increased small dense low-density lipoprotein (sdLDL), which differs from what has been observed in the nondiabetic population [5, 6]. Furthermore, despite attaining the target for low-density lipoprotein cholesterol (LDL-C), patients with diabetes still experience vascular complications [7], suggesting that the traditional use of a single lipid indicator (LDL-C) is no longer sufficient for elucidating vascular damage in individuals with diabetes. Hence, the exploration of a comprehensive blood lipid profile for improved prediction and assessment of diabetic vascular complications warrants attention.

The atherogenic index of plasma (AIP) was introduced to assess the atherosclerosis burden [8, 9]. Numerous studies have reported that AIP serves as a novel predictive indicator for cardiovascular diseases, regardless of conventional factors [10–13]. Moreover, AIP could predict insulin resistance [14], prediabetes [15], and diabetes onset [16–18]. In a second analysis conducted by Fu et al., it was revealed that AIP could function as a predictor of cardiovascular events in individuals with T2D [19]. Considering the coexistence of micro- and macrovascular complications in diabetes, questions have arisen as to whether AIP contributes to the incidence of microvascular diseases, including diabetic kidney disease (DKD) and diabetic retinopathy (DR). Until now, several cross-sectional studies have investigated the correlations of AIP with microvascular complications in T2D [20–23]; however, those studies yielded inconsistent results, which prompted us to investigate these relationships in a large prospective cohort.

Furthermore, there has been growing interest in investigating the correlation between individuals’ longitudinal trajectories of lipid indices and outcomes. Yan et al. reported that higher trajectories of lipid accumulation product (LAP) had a strong impact on the incidence of T2D beyond baseline LAP measurements [24]. Another study revealed significant correlations between HDL-C and TG trajectories and the onset of T2D [25]. Nevertheless, long-term AIP trajectories, which could indicate the prolonged influence of lipid overaccumulation, have not been previously published in diabetic complications; in particular, the associations of these trajectories with DKD and DR are unknown.

As few large longitudinal cohort studies have examined the relationship of AIP levels with microvascular complications in participants with T2D, the current study was to explore whether the AIP could be a predictive biomarker of DKD and DR and to assess the impact of the long-term trajectories of AIP across multiple follow-up visits on adverse outcomes.

## Methods

### Participants

T2D participants were recruited from the National Metabolic Management Center (MMC) at Ruijin Hospital, Shanghai Jiao Tong University School of Medicine [26–28]. A total of 4831 T2D participants with either DKD or DR follow-up data were included between June 2017 and June 2023. Generally, participants were recommended to undergo DKD testing every six months and DR examinations every year at the Ruijin center. Individuals without DKD follow-up data (*n* = 255), diagnosed with DKD at baseline (*n* = 1445), without baseline AIP data (*n* = 13), or with less than six months of follow-up (*n* = 175) were excluded from the survival analysis dataset for DKD (*n* = 2943). Furthermore, after excluding those with fewer than three AIP measurements, 2652 participants were included in the construction of the long-term AIP trajectory for DKD analysis. Similarly, for DR analysis, after excluding those without DR follow-up data (*n* = 1213), those diagnosed with referable DR at baseline (*n* = 220), those lacking baseline AIP data (*n* = 34), those with less than six months of follow-up (*n* = 4), and those with less than three AIP measurements(*n* = 190), this study enrolled 3360 T2D participants for DR survival analysis and 3170 for trajectory construction. A flowchart is displayed in Supplemental Fig. 1. The study protocol received approval from the Ruijin Hospital Ethics Committee. All participants signed informed consent before enrolment.

### Clinical data collection

At the MMC, participants generally underwent clinical examinations and completed standardized questionnaires at each visit. The recorded information was stored in an electronic medical system exclusively designed for MMC. Baseline covariates including sex, age, ideal smoking, diabetes duration, drinking status, body mass index (BMI), glycated haemoglobin (HbA1c), systolic blood pressure (SBP), and LDL-C were retrieved from the MMC. In this project, people who never smoked or successfully stopped smoking for over one year were classified as ‘ideal smoking’ status. More elaborate definitions and collection methods for covariates were provided in previous publications [26, 29].

### AIP at baseline and follow-up visits

The AIP was calculated as the logarithm (base 10) of the ratio of TG (mmol/L) to HDL-C (mmol/L). According to the standard operating procedures of MMC, participants were advised to visit the Ruijin center two to four times a year [30]. TG and HDL-C measurements were routinely taken at each visit, allowing us to obtain AIP values at baseline and follow-up visits. Participants who had at least three AIP measurements during the follow-up were selected for the construction of the AIP trajectory, and the number of AIP measurements for each participant varied between 3 and 21, averaging 7 measurements.

### Definition of DKD and DR

DKD was defined as an estimated glomerular filtration rate < 60 mL/min/1.73m^2^, a urinary albumin/creatinine ratio ≥ 3.39 mg/mmol, or both. The incidence of DR during follow-up in this study was defined as the onset of referable DR (moderate nonproliferative DR or worse) by fundus photography [31].

### Statistical analysis

Characteristics at baseline are presented as the mean ± standard deviation (SD) or median (interquartile range [IQR]) for continuous variables and as the number (proportion) for categorical variables. Comparisons between the participants who developed outcomes and those who did not were conducted using the Student’s t-test, the Wilcoxon rank-sum test, or the Chi-square test. Missing baseline covariates were imputed through multiple imputation.

Kaplan-Meier curves were plotted according to baseline AIP tertiles, followed by comparison using the log-rank test. Cox regression models were employed to assess the associations between baseline AIP and the risk of DKD and DR. Three models were stepwise adjusted for covariates: Model 1 was an unadjusted model; Model 2 was adjusted for age, sex, HbA1c, diabetes duration, BMI, SBP, ideal smoking, and drinking status; and Model 3 was additionally adjusted for LDL-C. To examine the potential nonlinear relationship between outcome incidence and baseline AIP levels, the current study plotted restricted cubic splines with three knots, adjusting for all confounders.

Group-based trajectory modelling was conducted to discern distinct longitudinal AIP value patterns during the follow-up visits, testing models with groups spanning from 2 to 5 [32]. The number of groups and the optimal trajectory shape (linear, quadratic, or cubic) were determined by the Bayesian information criterion and model interpretability. Each group comprised over 2% of the total population, and the average posterior probability of membership exceeded 70%. In the end, the best-fitting model consisted of three distinct trajectories. Subsequently, logistic regressions were applied to ascertain the influence of AIP trajectory groups on the onset of outcomes. The data were analysed with R software (version 4.1.2) and Stata 17.0. *P* < 0.05 was considered significant.

## Results

### Characteristics of study participants

During a median (IQR) follow-up time of 24.2 (26.3) months for DKD and 25.7 (27.0) months for DR, 709 of 2943 participants experienced new-onset DKD, and 193 of 3360 participants developed referable DR. The baseline clinical characteristics of the participants are presented based on outcome status (Table 1). Notably, the baseline AIP was higher in participants who developed DKD than in those who did not (*P* = 0.001). Compared with participants without incident DKD during follow-up, those who developed DKD appeared to be more women, older, with a longer diabetes duration, and higher BMI, SBP, and TG levels (all *P* < 0.05). For the analysis of DR, participants with DR development tended to be older, have higher HbA1c and SBP levels, have a lower BMI, and have a longer diabetes duration than those without DR (all *P* < 0.05).

**Table 1:** Baseline characteristics of the study participants according to outcome status

Characteristics	Overall	DKD -	DKD +	*P* value	Overall	DR -	DR +	*P* value
N	2943	2234	709		3360	3167	193	
Age (year)	55.18 ± 11.69	54.23 ± 11.56	58.17 ± 11.59	< 0.001	55.72 ± 11.59	55.55 ± 11.66	58.42 ± 10.08	< 0.001
Male, n (%)	1775 (60.31%)	1392 (62.31%)	383 (54.02%)	< 0.001	2008 (59.76%)	1881 (59.39%)	127 (65.80%)	0.092
HbA1c (%)	7.70 ± 1.68	7.70 ± 1.70	7.70 ± 1.59	0.974	7.75 ± 1.67	7.71 ± 1.65	8.46 ± 1.76	< 0.001
Diabetes duration (months)	88.72 ± 88.41	83.91 ± 85.48	103.72 ± 95.50	< 0.001	91.26 ± 87.80	87.81 ± 85.71	147.94 ± 101.5	< 0.001
BMI (kg/m^2^)	25.55 ± 3.87	25.38 ± 3.89	26.07 ± 3.76	< 0.001	25.83 ± 3.92	25.86 ± 3.96	25.34 ± 3.12	0.029
SBP (mmHg)	126.66 ± 16.55	125.31 ± 16.35	130.90 ± 16.49	< 0.001	128.59 ± 17.10	128.37 ± 17.05	132.14 ± 17.65	0.003
DBP (mmHg)	73.65 ± 10.38	73.59 ± 10.19	73.82 ± 10.94	0.626	74.57 ± 10.60	74.61 ± 10.62	74.02 ± 10.32	0.455
LDL-C (mmol/L)	3.05 ± 0.97	3.06 ± 0.97	2.99 ± 0.97	0.085	3.05 ± 0.96	3.05 ± 0.96	3.00 ± 0.95	0.470
HDL-C (mmol/L)	1.26 ± 0.32	1.26 ± 0.32	1.24 ± 0.31	0.072	1.25 ± 0.31	1.25 ± 0.31	1.27 ± 0.32	0.233
TG (mmol/L)	1.46 (1.03;2.10)	1.44 (1.00;2.09)	1.55 (1.12;2.21)	0.001	1.52 (1.08;2.21)	1.52 (1.08;2.22)	1.53 (1.05;2.10)	0.890
AIP	0.08 (-0.12;0.28)	0.07 (-0.13;0.27)	0.12 (-0.07;0.30)	0.001	0.10 (-0.09;0.30)	0.10 (-0.09;0.30)	0.10 (-0.10;0.29)	0.629
Ideal smoking, n (%)	2236 (77.02%)	1659 (75.44%)	577 (81.96%)	< 0.001	2600 (77.80%)	2455 (77.96%)	145 (75.13%)	0.407
Drinking, n (%)	284 (9.79%)	222 (10.10%)	62 (8.81%)	0.349	321 (9.61%)	295 (9.37%)	26 (13.47%)	0.080

Unimputed data are presented as mean ± SD, median (interquartile range), or n (%). DR refers to referable DR. Statistical comparisons between the participants who developed outcomes and those who did not were conducted using the Student’s t-test for means, the Wilcoxon rank-sum test for medians, or the Chi-square test for proportions. Abbreviations: *HbA1c* glycated haemoglobin, *BMI* body mass index, *SBP* systolic blood pressure, *DBP* diastolic blood pressure, *LDL-C* low-density lipoprotein cholesterol, *HDL-C* high-density lipoprotein cholesterol, *TG* Triglyceride, *AIP* atherogenic index of plasma, *DKD* diabetic kidney disease, *DR* diabetic retinopathy

### Baseline AIP was linked to the incidence of DKD rather than DR

**Fig. 1:**
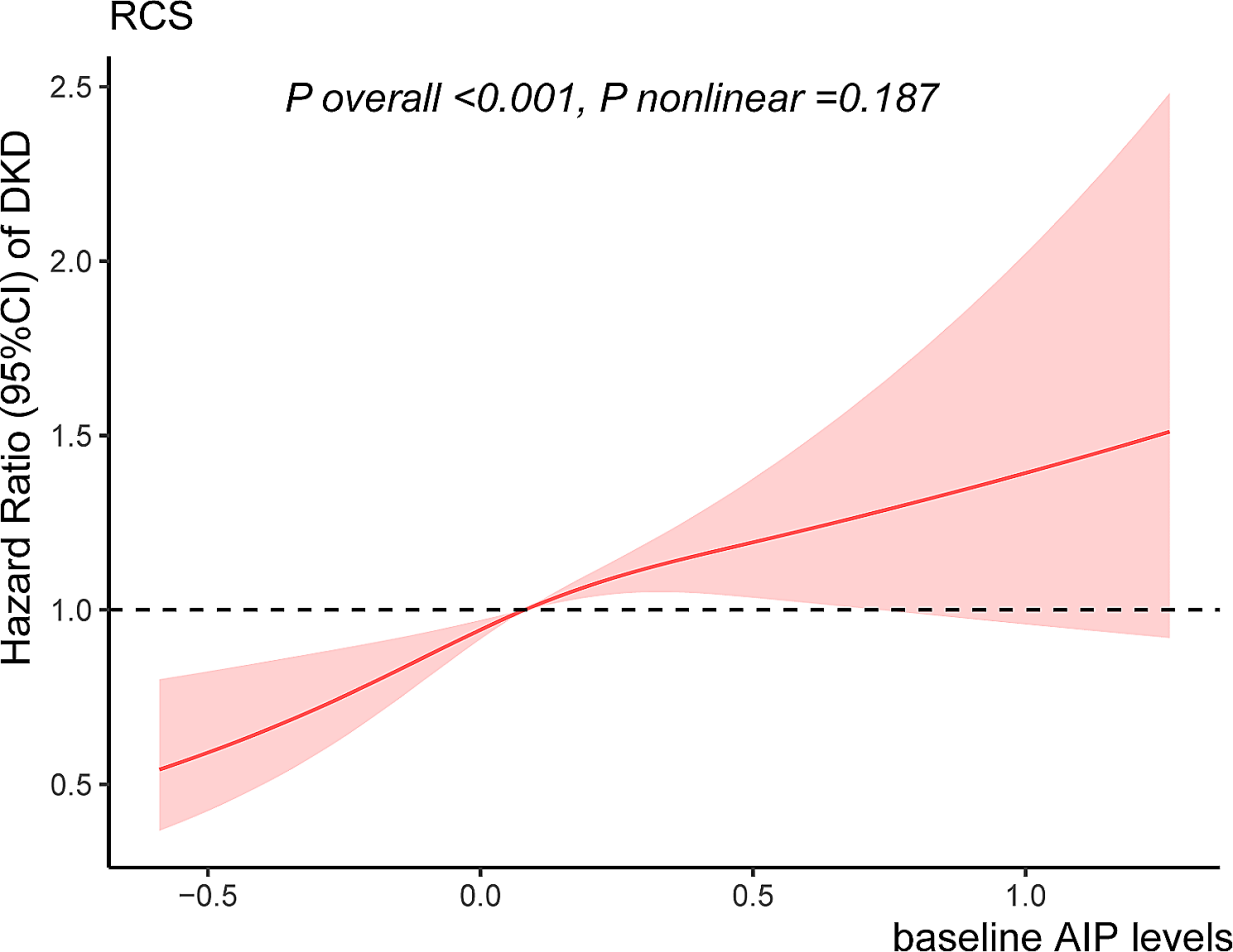
Restricted cubic spline regression for baseline AIP levels and DKD. Adjusted for age, sex, HbA1c, duration of diabetes, BMI, SBP, ideal smoking, drinking, and LDL-C. The solid line indicates the smoothed fitted relationship and the red shaded area represents 95% CI. Abbreviations: *AIP* atherogenic index of plasma, *DKD* diabetic kidney disease, *CI* confidence interval

Kaplan-Meier analyses revealed that individuals in tertile 3 of AIP had the highest risk of DKD incidence (log-rank *P* < 0.001), as presented in Supplemental Fig. 2. Table 2 presents the hazard ratio (HR) and 95% confidence interval (CI) obtained from Cox regression analyses, elucidating the relationships of baseline AIP with the risk of DKD and DR. Multivariate-adjusted models indicated that each one-unit rise in baseline AIP was correlated with a 75% elevated probability (HR 1.75; 95% CI 1.36–2.26) of DKD. When baseline AIP were stratified into tertiles and examined as a categorical variable, similar results were detected. Specifically, tertile 3 (HR 1.47; 95% CI 1.21–1.79) and tertile 2 (HR 1.27; 95% CI 1.05–1.54) of AIP presented a greater risk of incident DKD when using tertile 1 as the reference. Restricted cubic spline regression revealed a linear and positive relationship between baseline AIP and the incidence of DKD (*P* nonlinear = 0.187, *P* overall < 0.001) (Fig. 1). Moreover, no correlation was observed between DR and baseline AIP, which was considered either a continuous or categorical variable, in the three models.

**Table 2:** Association of AIP level at baseline with the incidence of DKD and DR

		Model 1		Model 2		Model 3	
	Cases/N	HR (95% CI)	*P* value	HR (95% CI)	*P* value	HR (95% CI)	*P* value
DKD							
AIP (per unit)	709/2943	1.61(1.28,2.04)	< 0.001	1.75(1.35,2.26)	< 0.001	1.75(1.36,2.26)	< 0.001
Tertile 1 (< -0.05)	201/981	Reference		Reference		Reference	
Tertile 2 (-0.05, 0.21)	248/981	1.31(1.09,1.57)	0.005	1.25(1.03,1.51)	0.024	1.27(1.05,1.54)	0.016
Tertile 3 (≥ 0.21)	260/981	1.43(1.19,1.73)	< 0.001	1.45(1.20,1.76)	< 0.001	1.47(1.21,1.79)	< 0.001
DR							
AIP (per unit)	193/3360	0.90(0.57,1.42)	0.660	0.91(0.56,1.49)	0.717	0.92(0.57,1.49)	0.722
Tertile 1 (< -0.02)	66/1120	Reference		Reference		Reference	
Tertile 2 (-0.02, 0.23)	64/1120	1.00(0.71,1.41)	0.998	1.04(0.73,1.48)	0.840	1.05(0.74,1.51)	0.774
Tertile 3 (≥ 0.23)	63/1120	1.00(0.71,1.41)	0.995	1.04(0.72,1.50)	0.845	1.05(0.72,1.52)	0.810

Model 1: Crude model; Model 2: Adjusted for age, sex, HbA1c, duration of diabetes, BMI, SBP, ideal smoking, drinking; Model 3: Adjusted for covariables in model 2 plus LDL-C. DR refers to referable DR. Abbreviations: *HR* Hazard ratio, *CI* Confidence interval, *AIP* atherogenic index of plasma, *DKD* diabetic kidney disease, *DR* diabetic retinopathy

### AIP trajectories were related to incident DKD but not DR

**Fig. 2:**
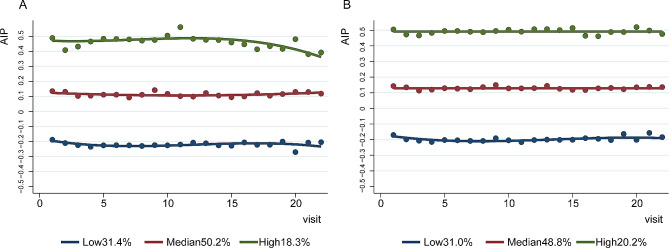
Trajectories of AIP during follow-up visits in the DKD (**A**) and DR (**B**) longitudinal analysis datasets. DR refers to referable DR. Abbreviations: *AIP* atherogenic index of plasma, *DKD* diabetic kidney disease, *DR* diabetic retinopathy

After excluding those with fewer than three recorded AIP visits, a cohort of 2652 participants with T2D was included in the group-based trajectory modelling in the DKD longitudinal dataset (Supplemental Fig. 1). This model delineated three distinct trajectory classes: Low (834 participants, 31.4%), Median (1332 participants, 50.2%), and High (486 participants, 18.3%), each exhibiting stable AIP levels throughout the follow-up period (Fig. 2A). Table 3 displays the odds ratio (OR) and 95% CI of AIP trajectories for the occurrence of DKD, as determined through logistic regression models. With respect to the Low trajectory, the Median and High AIP trajectories presented 69% (95% CI: 1.36–2.10) and 74% (95% CI 1.33–2.28) higher odds of developing DKD, respectively. After controlling for baseline covariates in Model 3, the Median and High trajectories exhibited odds of DKD incidence that were 1.84-fold (95% CI 1.46–2.32) and 2.17-fold (95% CI 1.62–2.91), respectively. Regarding trajectories of AIP levels over the follow-up visits and the results of logistic regression analyses for DR (Fig. 2B; Table 3), various AIP trajectories exhibited no significant differences in the occurrence of DR (all *P* > 0.05).

**Table 3:** OR and 95% CI of AIP Trajectories on DKD and DR in the T2D participants

AIP Trajectory	N (%)	Model 1		Model 2		Model 3		
		OR (95% CI)	*P*	OR (95% CI)	*P*	OR (95% CI)		*P*
DKD								
Low	834(31.4%)	Reference		Reference		Reference		
Median	1332(50.2%)	1.69(1.36,2.10)	< 0.001	1.80(1.44,2.27)	< 0.001	1.84(1.46,2.32)	< 0.001	
High	486(18.3%)	1.74(1.33,2.28)	< 0.001	2.18(1.63,2.93)	< 0.001	2.17(1.62,2.91)	< 0.001	
DR								
Low	982(31.0%)	Reference		Reference		Reference		
Median	1547(48.8%)	1.01(0.72,1.42)	0.966	1.08(0.75,1.55)	0.691	1.09(0.76,1.57)	0.653	
High	641(20.2%)	0.78(0.49,1.22)	0.287	0.85(0.51,1.38)	0.515	0.84(0.51,1.37)	0.496	

Model 1: Crude model; Model 2: Adjusted for age, sex, HbA1c, duration of diabetes, BMI, SBP, ideal smoking, drinking; Model 3: Adjusted for covariables in model 2 plus LDL-C. DR refers to referable DR. Abbreviations: *OR* Odds ratio, *CI* Confidence interval, *AIP* atherogenic index of plasma, *DKD* diabetic kidney disease, *DR* diabetic retinopathy, *T2D* type 2 diabetes

## Discussion

This investigation revealed that elevated baseline AIP was linked to an increased risk of DKD but not DR. The correlation between baseline AIP and DKD risk followed a positive and linear dose-response association. Moreover, the present study identified three distinct trajectories of AIP with repeated measurements of this novel index across the follow-up visits, which showed that the High and Median AIP trajectories carried higher odds of incident DKD. These findings indicated that high levels of AIP could predict the occurrence of DKD.

The AIP, as a simple and easily accessible indicator, integrates HDL-C and TG concentrations, offering a more comprehensive representation of the pathogenesis and specific nature of dyslipidaemia compared to elevated TG or reduced HDL-C levels alone [33]. Some studies have shown that AIP could represent the quantity of sdLDL particles (subfraction of LDL-C) [34]. sdLDL, characterized by difficulty in being cleared from the circulation, susceptibility to oxidation, and ease of uptake by macrophages to form foam cells, contributes to an elevated risk of microvascular complications [35]. Nonetheless, the clinical applicability of sdLDL is restricted because of its intricate and expensive measurement process [13]. Thus, the novel lipid indicator AIP might function as a more effective marker for evaluating vascular risk.

Unlike the findings of prior research revealing that AIP was associated with macrovascular events [19], the present investigation focused primarily on examining the relationships between AIP and microvascular diseases in the T2D population. Previous studies investigating the associations between AIP and kidney damage in individuals with T2D were cross-sectional, revealing inconsistent results, and longitudinal cohort studies were scarce. For instance, several retrospective studies reported that an increased AIP was linked to the development of microalbuminuria in 4358 and 335 Chinese T2D patients, respectively [20, 22]. However, in a study involving 2523 patients with T2D, no significant difference in the diabetic nephropathy prevalence, determined by urinary microalbumin, was observed among AIP tertiles [21]. Hence, the current study utilized a large longitudinal cohort to further provide evidence that baseline AIP was not only correlated with the incidence of DKD but also exhibited a positive and linear relationship. The present results underscored the predictive value of AIP for DKD, independent of LDL-C.

Indeed, both DR and DKD are diabetic microvascular complications, but the current study found no association between AIP, either at baseline or in the trajectories, and the occurrence of DR. This phenomenon might be explained by the different influences of circulating lipids on the initiation of kidney and eye damage in individuals with diabetes [36]. Epidemiological studies have indicated that the links between lipid profiles and retinopathy are not as strong as those observed for kidney disease [36–38]. In contrast to the kidney, the blood-retinal barrier’s integrity shields the retina from possible harm caused by plasma lipoproteins [39], and the regulation or dysregulation of intraretinal lipid transport may be more crucial in the development of DR than plasma lipid levels, which possibly explains the absence of a correlation between AIP and DR. Further multicenter clinical investigations are warranted to confirm the correlation between AIP and the onset of DR.

Considering the dynamic changes in the AIP over time, prior research relying on a single AIP measurement at baseline could not accurately capture long-term exposure. Based on the MMC medical system, researchers collected multiple AIP measurements at different visits, thereby enabling the identification of long-term AIP trajectories in a real-world study. Importantly, assessing the effect of AIP trajectories on the occurrence of DKD and DR provided more reliable and robust results than analysing only the impact of baseline AIP levels. This investigation was the first to elucidate the influence of long-term trends in the AIP on the incidence of DKD and DR in participants with T2D, and these results provided insights into the cumulative load of AIP for DKD incidence. In the clinical setting, graphical shapes of AIP values during follow-up visits could be plotted to discern T2D participants with similarly higher AIP trajectories. It is likely that the corresponding group could benefit from more frequent screening and more intensive management of DKD.

The progression of DKD is intricate and involves complex interactions among factors such as oxidative stress, endothelial dysfunction, and abnormal cytokine and growth factor production [40]. However, the mechanism of the relationship between AIP and incident DKD is unclear. One possible explanation was that AIP was calculated from TG and HDL, and DKD development was correlated with reduced HDL-C and elevated TG [41]. HDL particles might offer protection in the process, including the transport of cholesterol in the reverse direction, along with exerting antioxidant and anti-inflammatory effects [42]. HDL-C also has the potential to improve glycaemic regulation by influencing glucose absorption in skeletal muscle [43] and safeguarding islet β-cell function [44]. These defensive functions of HDL particles appear to be impaired in the context of diabetes. On the other hand, people who did not have T2D but had high TG levels seldom developed kidney disease, illustrating that dyslipidaemia was not enough to cause kidney damage. Indeed, the metabolic dysfunction characteristics of diabetes (hyperglycaemia, insulin resistance) potentially play a crucial role in facilitating the lipotoxic effects on the microvascular network [41]. The kidney is particularly susceptible to lipotoxic damage, which is mediated by factors such as malfunctioning adipose tissue, insulin resistance, elevated plasma nonesterified fatty acids, and obesity [45].

### Study strengths and limitations

First, this study was the first longitudinal design to examine the value of AIP as a predictor of microvascular diseases in T2D. Second, this cohort consisted of a relatively large sample size and included multiple follow-up visits. These enabled us to effectively monitor AIP trajectory patterns, ultimately bolstering the robustness and reliability of the findings.

On the other hand, several limitations should be considered. First, the current research was single-center, and additional validation in multiple centers is necessary. Second, as this study predominantly consisted of Chinese individuals with T2D, the generalizability of the findings to other races was limited. Although these limitations impact the clinical use of AIP as a predictor of DKD, this study underscores the significance of closely monitoring AIP in participants with T2D over the follow-up period.

## Conclusions

The current investigation indicates a linear correlation between elevated baseline AIP and a greater risk of developing DKD but not DR. The High and Median AIP trajectories, denoting prolonged exposure to elevated AIP levels, carry the greater odds of incident DKD. These results suggest that AIP may be a valuable predictor for evaluating the risk of developing DKD, rather than DR, in participants with T2D, which implies that regular screening and active management of AIP in diabetic patients may offer potential benefits in preventing the incidence of DKD.

### Electronic supplementary material

Below is the link to the electronic supplementary material.

Supplementary Material 1

## Data Availability

Due to the privacy concerns of the participants, the datasets of the current study are not publicly accessible, but are available from the corresponding author on reasonable request.

## Abbreviations

AIP: Atherogenic index of plasma
T2D: Type 2 diabetes
DKD: Diabetic kidney disease
DR: Diabetic retinopathy
TG: Triglycerides
HDL-C: High-density lipoprotein cholesterol
sdLDL: Small dense low-density lipoprotein
LDL-C: Low-density lipoprotein cholesterol
MMC: Metabolic Management Center
BMI: Body mass index
SBP: Systolic blood pressure
HbA1c: Glycated haemoglobin
SD: Standard deviation
IQR: Interquartile range
OR: Odds ratio
HR: Hazard ratio
CI: Confidence interval
