# Supplementary material for: Impact of baseline and trajectory of the atherogenic index of plasma on incident diabetic kidney disease and retinopathy in participants with type 2 diabetes: a longitudinal cohort study
Source: Lipids Health Dis. 2024 Jan 11;23:11. doi: 10.1186/s12944-024-02003-5 (PMC10782533; doi:10.1186/s12944-024-02003-5)
Supplement: Supplementary file 1 — Supplementary Material 1 [file 12944_2024_2003_MOESM1_ESM.docx]

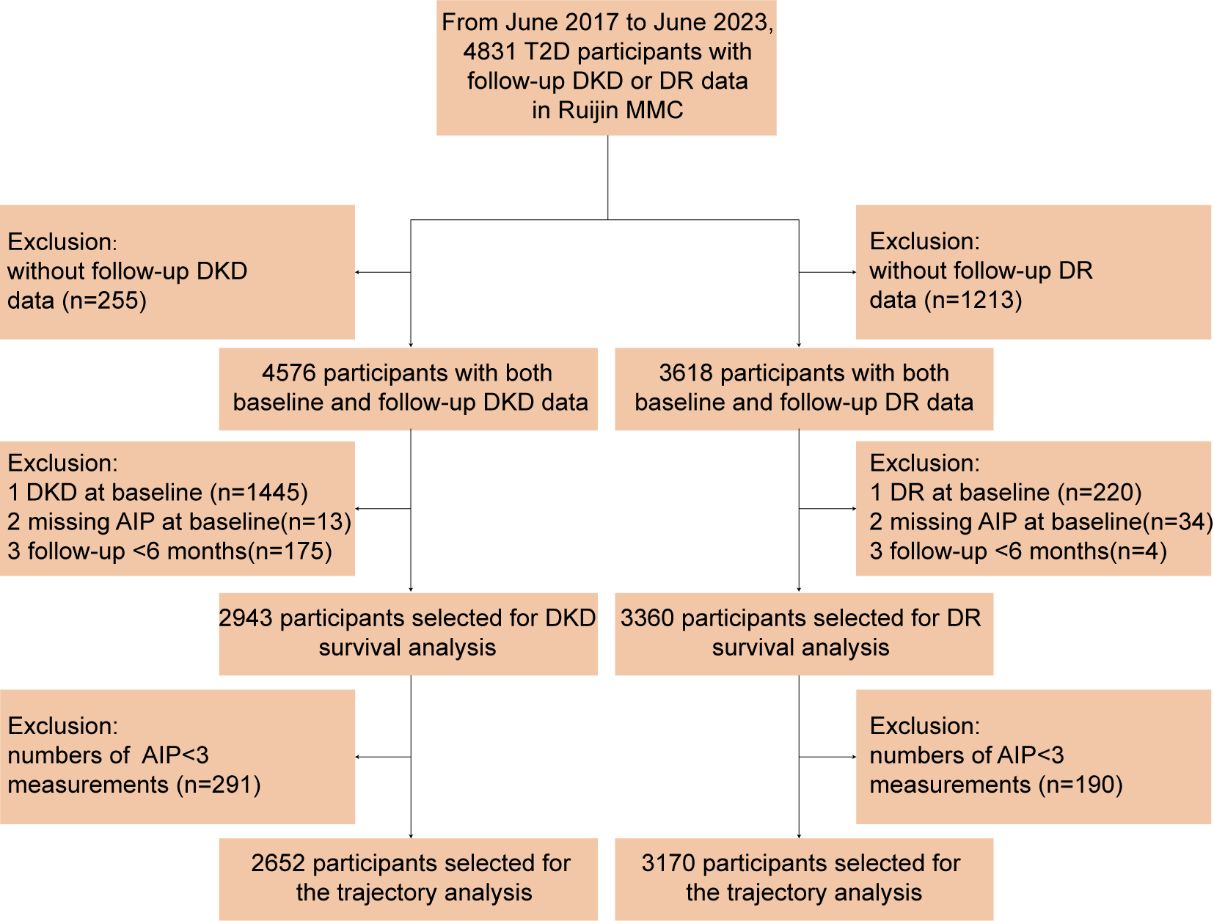


Supplemental Figure 1. Flowchart of inclusion and exclusion. DR refers to referable DR. Abbreviations: *MMC* Metabolic Management Center, *AIP* atherogenic index of plasma, *DKD* diabetic kidney disease, *DR* diabetic retinopathy


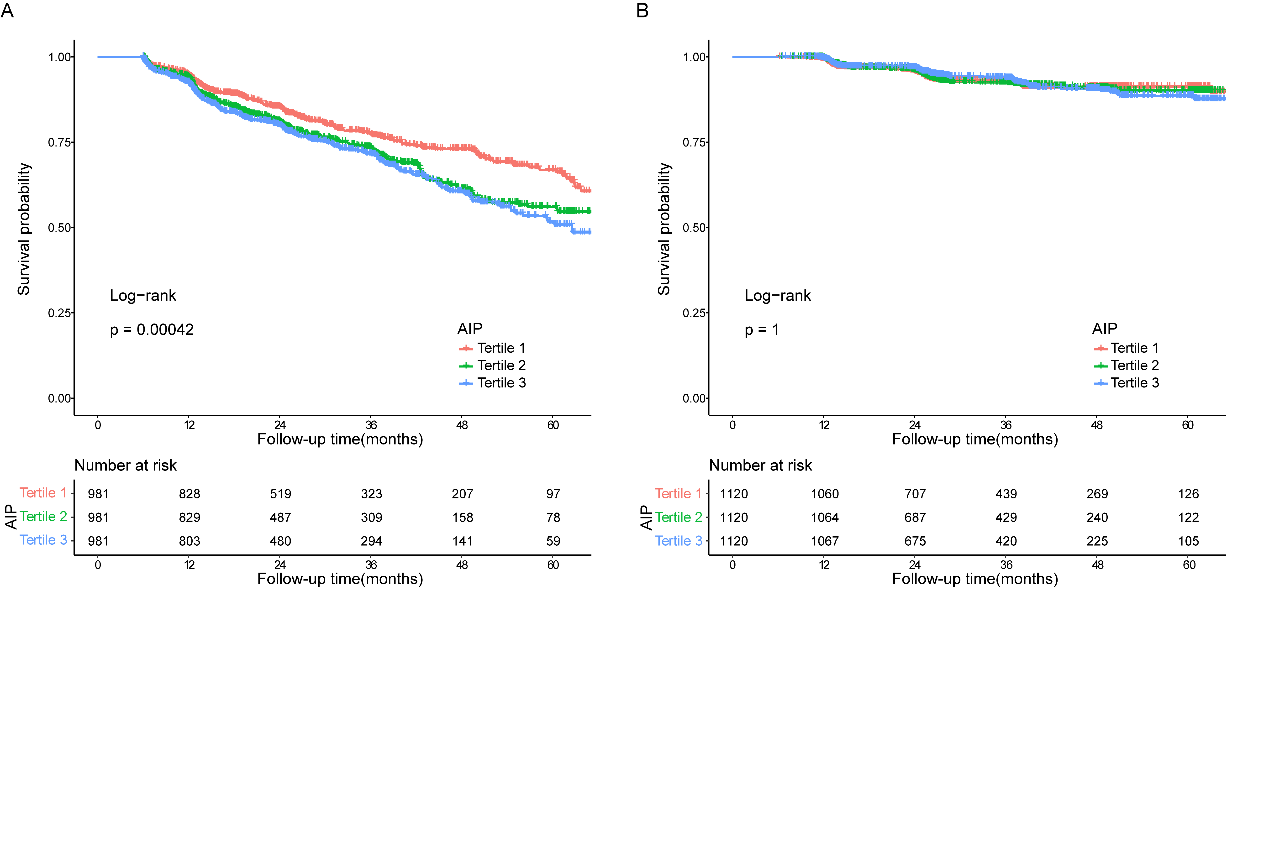


Supplemental Figure 2. Kaplan–Meier curves according to AIP tertiles for outcomes. (**A**) DKD; (**B**) DR

DR refers to referable DR. Abbreviations: *AIP* atherogenic index of plasma, *DKD* diabetic kidney disease, *DR* diabetic retinopathy
